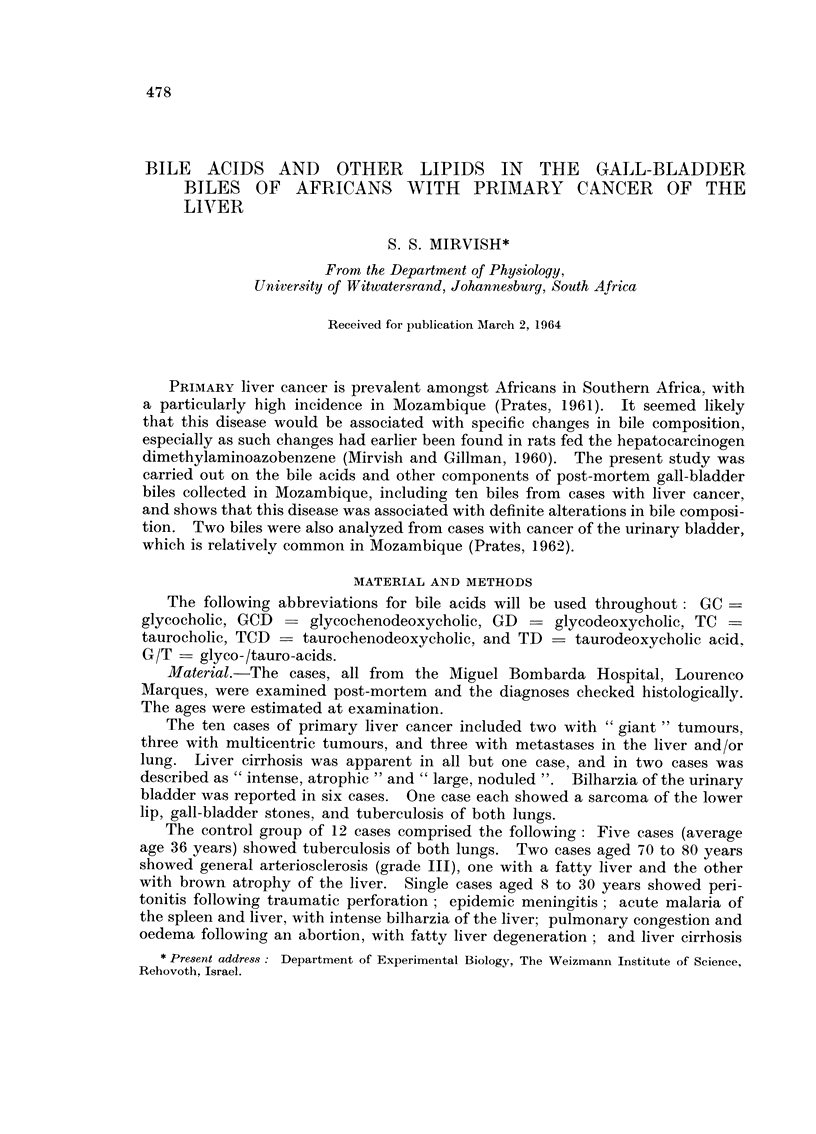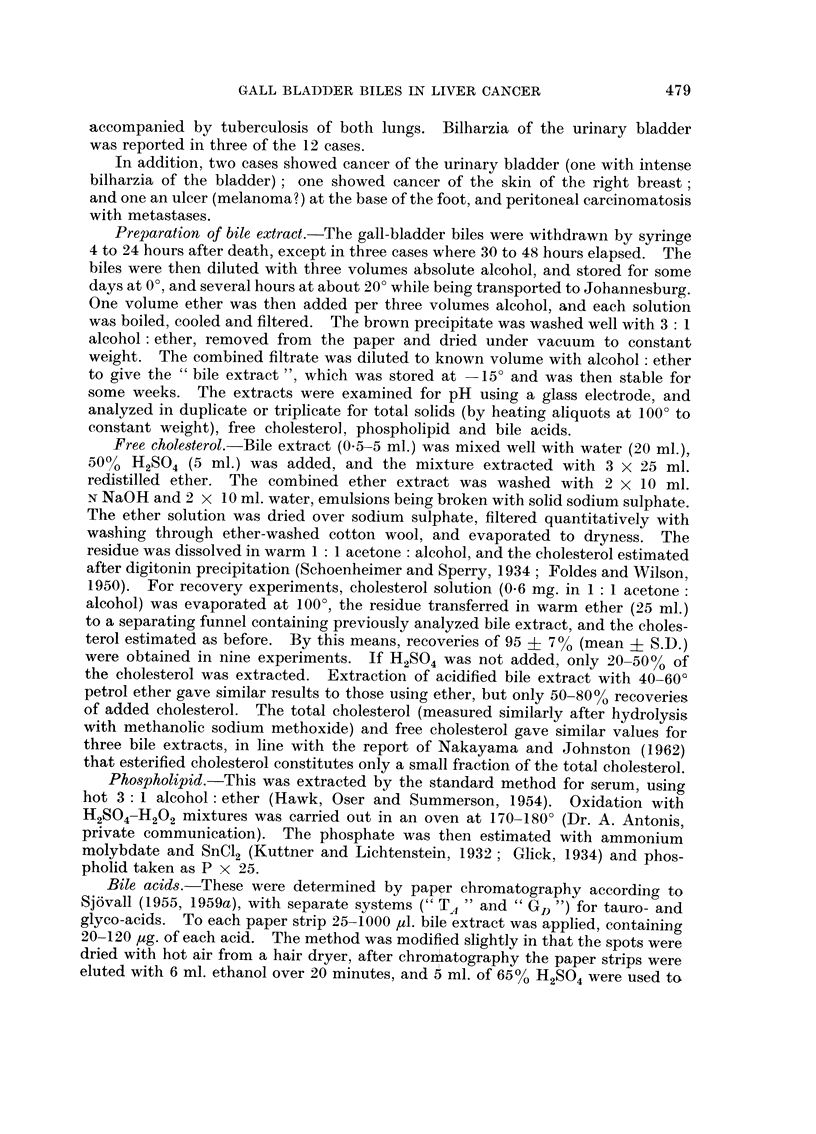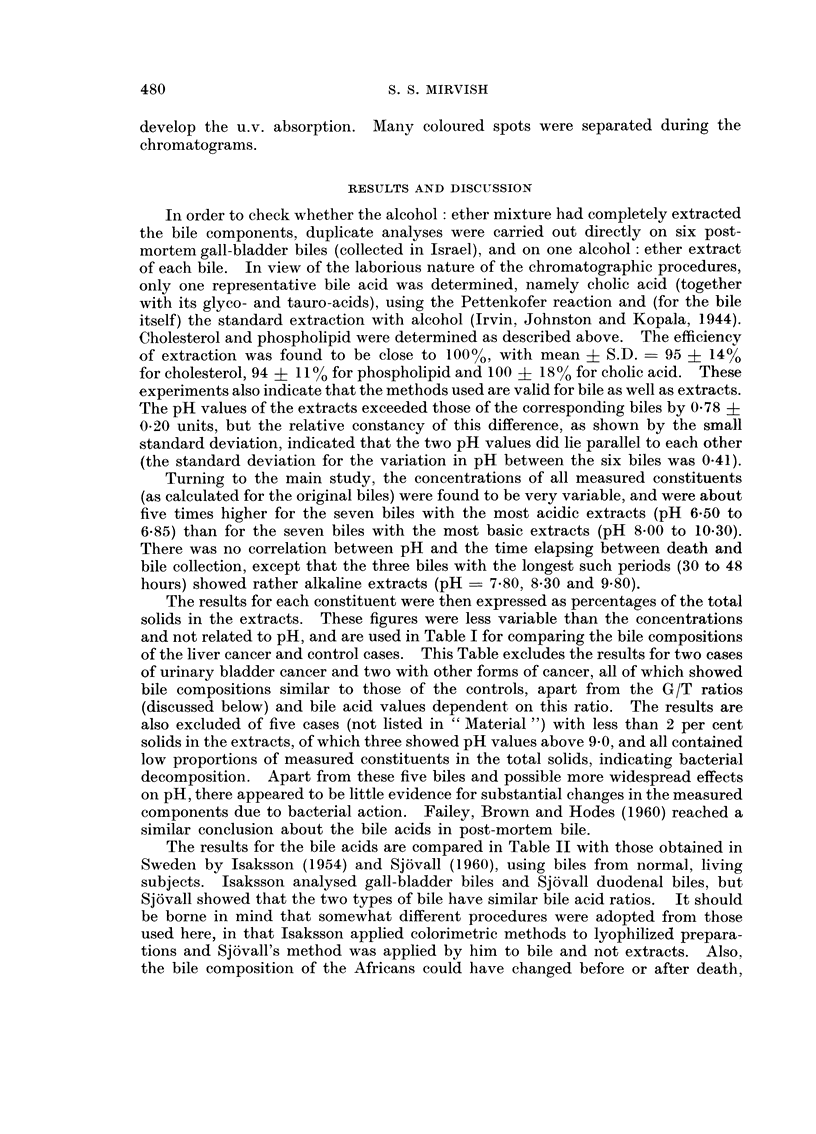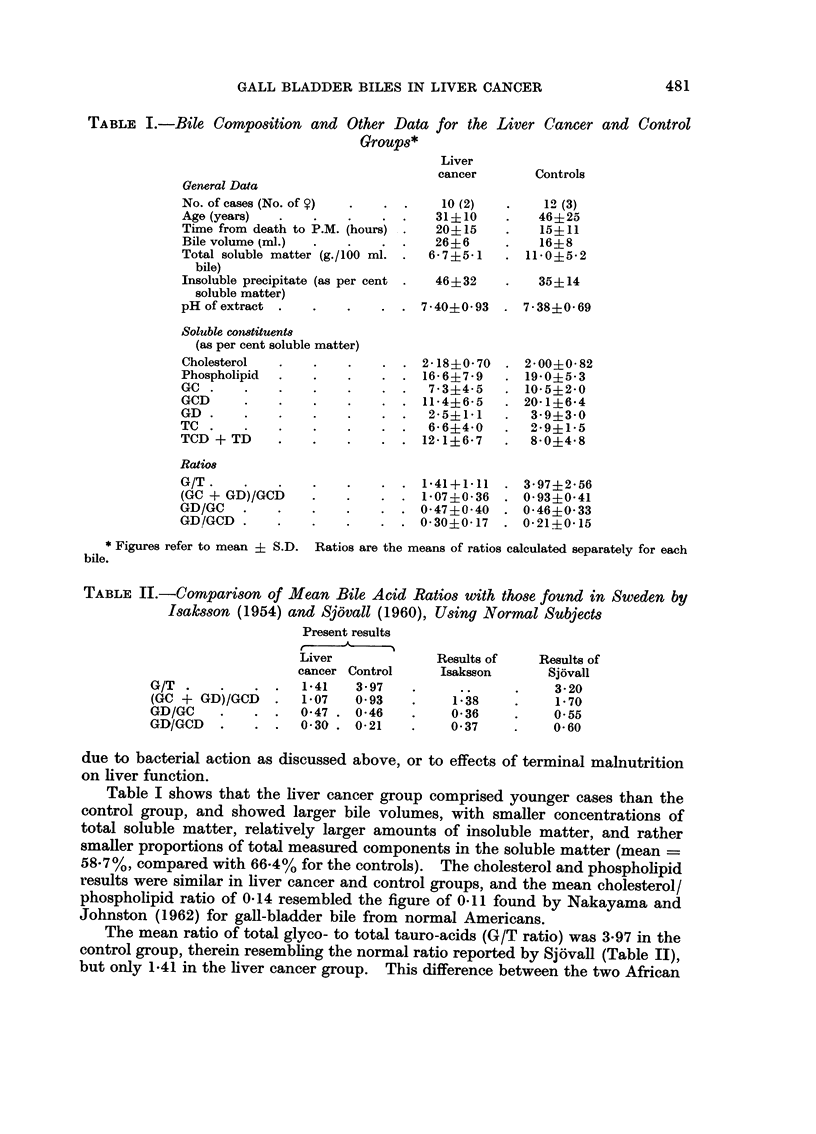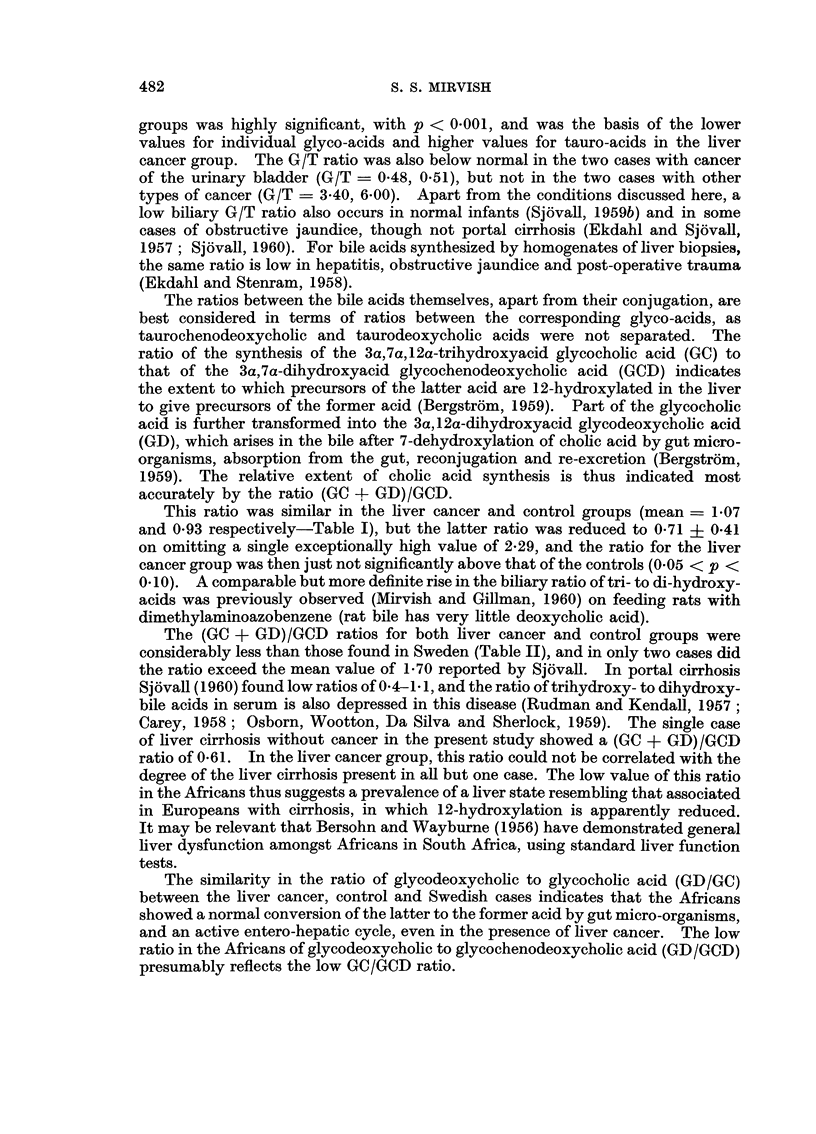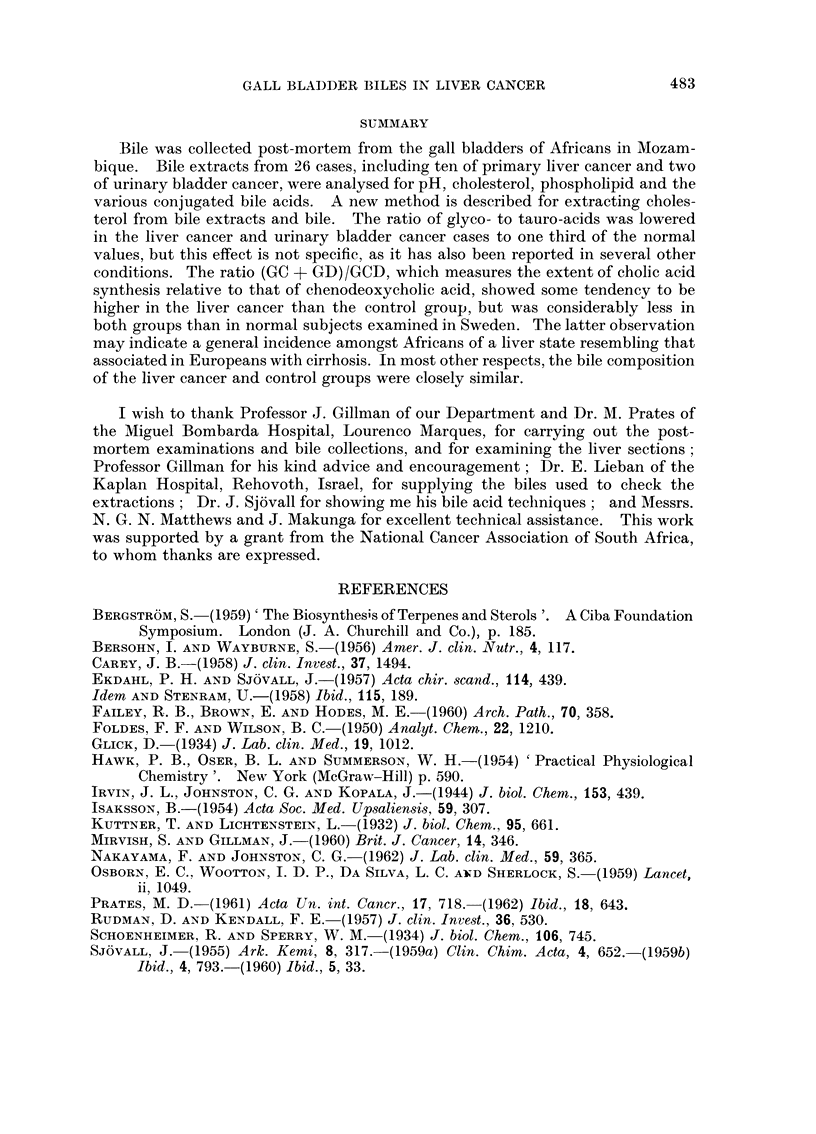# Bile Acids and Other Lipids in the Gall-Bladder Biles of Africans with Primary Cancer of the Liver

**DOI:** 10.1038/bjc.1964.53

**Published:** 1964-09

**Authors:** S. S. Mirvish


### BILE ACIDS AND OTHER LIPIDS IN THE GALL-BLADDER BILES OF AFRICANS WITH PRIMARY CANCER OF THE LIVER

S. S. MIRVISH*

*From the Department of Physiology, University of Witwatersrand, Johannesburg, South Africa*

Received for publication March 2, 1964

PRIMARY liver cancer is prevalent amongst Africans in Southern Africa, with a particularly high incidence in Mozambique (Prates, 1961). It seemed likely that this disease would be associated with specific changes in bile composition, especially as such changes had earlier been found in rats fed the hepatocarcinogen dimethylaminoazobenzene (Mirvish and Gillman, 1960). The present study was carried out on the bile acids and other components of post-mortem gall-bladder biles collected in Mozambique, including ten biles from cases with liver cancer, and shows that this disease was associated with definite alterations in bile composition. Two biles were also analyzed from cases with cancer of the urinary bladder, which is relatively common in Mozambique (Prates, 1962).

### MATERIAL AND METHODS

The following abbreviations for bile acids will be used throughout: GC = glycocholic, GCD = glycochenodeoxycholic, GD = glycodeoxycholic, TC = taurocholic, TCD = taurochenodeoxycholic, and TD = taurodeoxycholic acid, G/T = glyco-/tauro-acids.

*Material.*—The cases, all from the Miguel Bombarda Hospital, Lourenco Marques, were examined post-mortem and the diagnoses checked histologically. The ages were estimated at examination.

The ten cases of primary liver cancer included two with " giant " tumours, three with multicentric tumours, and three with metastases in the liver and/or lung. Liver cirrhosis was apparent in all but one case, and in two cases was described as " intense, atrophic " and " large, noduled ". Bilharzia of the urinary bladder was reported in six cases. One case each showed a sarcoma of the lower lip, gall-bladder stones, and tuberculosis of both lungs.

The control group of 12 cases comprised the following: Five cases (average age 36 years) showed tuberculosis of both lungs. Two cases aged 70 to 80 years showed general arteriosclerosis (grade III), one with a fatty liver and the other with brown atrophy of the liver. Single cases aged 8 to 30 years showed peritonitis following traumatic perforation; epidemic meningitis; acute malaria of the spleen and liver, with intense bilharzia of the liver; pulmonary congestion and oedema following an abortion, with fatty liver degeneration; and liver cirrhosis

* *Present address:* Department of Experimental Biology, The Weizmann Institute of Science, Rehovoth, Israel.

accompanied by tuberculosis of both lungs. Bilharzia of the urinary bladder was reported in three of the 12 cases.

In addition, two cases showed cancer of the urinary bladder (one with intense bilharzia of the bladder); one showed cancer of the skin of the right breast; and one an ulcer (melanoma?) at the base of the foot, and peritoneal carcinomatosis with metastases.

*Preparation of bile extract.*—The gall-bladder biles were withdrawn by syringe 4 to 24 hours after death, except in three cases where 30 to 48 hours elapsed. The biles were then diluted with three volumes absolute alcohol, and stored for some days at 0°, and several hours at about 20° while being transported to Johannesburg. One volume ether was then added per three volumes alcohol, and each solution was boiled, cooled and filtered. The brown precipitate was washed well with 3 : 1 alcohol : ether, removed from the paper and dried under vacuum to constant weight. The combined filtrate was diluted to known volume with alcohol : ether to give the " bile extract ", which was stored at −15° and was then stable for some weeks. The extracts were examined for pH using a glass electrode, and analyzed in duplicate or triplicate for total solids (by heating aliquots at 100° to constant weight), free cholesterol, phospholipid and bile acids.

*Free cholesterol.*—Bile extract (0·5–5 ml.) was mixed well with water (20 ml.), 50% $H_2SO_4$ (5 ml.) was added, and the mixture extracted with 3 × 25 ml. redistilled ether. The combined ether extract was washed with 2 × 10 ml. N NaOH and 2 × 10 ml. water, emulsions being broken with solid sodium sulphate. The ether solution was dried over sodium sulphate, filtered quantitatively with washing through ether-washed cotton wool, and evaporated to dryness. The residue was dissolved in warm 1 : 1 acetone : alcohol, and the cholesterol estimated after digitonin precipitation (Schoenheimer and Sperry, 1934; Foldes and Wilson, 1950). For recovery experiments, cholesterol solution (0·6 mg. in 1 : 1 acetone : alcohol) was evaporated at 100°, the residue transferred in warm ether (25 ml.) to a separating funnel containing previously analyzed bile extract, and the cholesterol estimated as before. By this means, recoveries of 95 ± 7% (mean ± S.D.) were obtained in nine experiments. If $H_2SO_4$ was not added, only 20–50% of the cholesterol was extracted. Extraction of acidified bile extract with 40–60° petrol ether gave similar results to those using ether, but only 50–80% recoveries of added cholesterol. The total cholesterol (measured similarly after hydrolysis with methanolic sodium methoxide) and free cholesterol gave similar values for three bile extracts, in line with the report of Nakayama and Johnston (1962) that esterified cholesterol constitutes only a small fraction of the total cholesterol.

*Phospholipid.*—This was extracted by the standard method for serum, using hot 3 : 1 alcohol : ether (Hawk, Oser and Summerson, 1954). Oxidation with $H_2SO_4$–$H_2O_2$ mixtures was carried out in an oven at 170–180° (Dr. A. Antonis, private communication). The phosphate was then estimated with ammonium molybdate and $SnCl_2$ (Kuttner and Lichtenstein, 1932; Glick, 1934) and phospholid taken as P × 25.

*Bile acids.*—These were determined by paper chromatography according to Sjövall (1955, 1959*a*), with separate systems (" $T_A$ " and " $G_D$ ") for tauro- and glyco-acids. To each paper strip 25–1000 μl. bile extract was applied, containing 20–120 μg. of each acid. The method was modified slightly in that the spots were dried with hot air from a hair dryer, after chromatography the paper strips were eluted with 6 ml. ethanol over 20 minutes, and 5 ml. of 65% $H_2SO_4$ were used to develop the u.v. absorption. Many coloured spots were separated during the chromatograms.

### RESULTS AND DISCUSSION

In order to check whether the alcohol : ether mixture had completely extracted the bile components, duplicate analyses were carried out directly on six post-mortem gall-bladder biles (collected in Israel), and on one alcohol : ether extract of each bile. In view of the laborious nature of the chromatographic procedures, only one representative bile acid was determined, namely cholic acid (together with its glyco- and tauro-acids), using the Pettenkofer reaction and (for the bile itself) the standard extraction with alcohol (Irvin, Johnston and Kopala, 1944). Cholesterol and phospholipid were determined as described above. The efficiency of extraction was found to be close to 100%, with mean $\pm$ S.D. = 95 $\pm$ 14% for cholesterol, 94 $\pm$ 11% for phospholipid and 100 $\pm$ 18% for cholic acid. These experiments also indicate that the methods used are valid for bile as well as extracts. The pH values of the extracts exceeded those of the corresponding biles by 0·78 $\pm$ 0·20 units, but the relative constancy of this difference, as shown by the small standard deviation, indicated that the two pH values did lie parallel to each other (the standard deviation for the variation in pH between the six biles was 0·41).

Turning to the main study, the concentrations of all measured constituents (as calculated for the original biles) were found to be very variable, and were about five times higher for the seven biles with the most acidic extracts (pH 6·50 to 6·85) than for the seven biles with the most basic extracts (pH 8·00 to 10·30). There was no correlation between pH and the time elapsing between death and bile collection, except that the three biles with the longest such periods (30 to 48 hours) showed rather alkaline extracts (pH = 7·80, 8·30 and 9·80).

The results for each constituent were then expressed as percentages of the total solids in the extracts. These figures were less variable than the concentrations and not related to pH, and are used in Table I for comparing the bile compositions of the liver cancer and control cases. This Table excludes the results for two cases of urinary bladder cancer and two with other forms of cancer, all of which showed bile compositions similar to those of the controls, apart from the G/T ratios (discussed below) and bile acid values dependent on this ratio. The results are also excluded of five cases (not listed in " Material ") with less than 2 per cent solids in the extracts, of which three showed pH values above 9·0, and all contained low proportions of measured constituents in the total solids, indicating bacterial decomposition. Apart from these five biles and possible more widespread effects on pH, there appeared to be little evidence for substantial changes in the measured components due to bacterial action. Failey, Brown and Hodes (1960) reached a similar conclusion about the bile acids in post-mortem bile.

The results for the bile acids are compared in Table II with those obtained in Sweden by Isaksson (1954) and Sjövall (1960), using biles from normal, living subjects. Isaksson analysed gall-bladder biles and Sjövall duodenal biles, but Sjövall showed that the two types of bile have similar bile acid ratios. It should be borne in mind that somewhat different procedures were adopted from those used here, in that Isaksson applied colorimetric methods to lyophilized preparations and Sjövall's method was applied by him to bile and not extracts. Also, the bile composition of the Africans could have changed before or after death,

TABLE I.—*Bile Composition and Other Data for the Liver Cancer and Control Groups**

| | Liver cancer | Controls |
|---|---|---|
| *General Data* | | |
| No. of cases (No. of ♀) | 10 (2) | 12 (3) |
| Age (years) | 31±10 | 46±25 |
| Time from death to P.M. (hours) | 20±15 | 15±11 |
| Bile volume (ml.) | 26±6 | 16±8 |
| Total soluble matter (g./100 ml. bile) | 6·7±5·1 | 11·0±5·2 |
| Insoluble precipitate (as per cent soluble matter) | 46±32 | 35±14 |
| pH of extract | 7·40±0·93 | 7·38±0·69 |
| *Soluble constituents* (as per cent soluble matter) | | |
| Cholesterol | 2·18±0·70 | 2·00±0·82 |
| Phospholipid | 16·6±7·9 | 19·0±5·3 |
| GC | 7·3±4·5 | 10·5±2·0 |
| GCD | 11·4±6·5 | 20·1±6·4 |
| GD | 2·5±1·1 | 3·9±3·0 |
| TC | 6·6±4·0 | 2·9±1·5 |
| TCD + TD | 12·1±6·7 | 8·0±4·8 |
| *Ratios* | | |
| G/T | 1·41+1·11 | 3·97±2·56 |
| (GC + GD)/GCD | 1·07±0·36 | 0·93±0·41 |
| GD/GC | 0·47±0·40 | 0·46±0·33 |
| GD/GCD | 0·30±0·17 | 0·21±0·15 |

* Figures refer to mean ± S.D. Ratios are the means of ratios calculated separately for each bile.

TABLE II.—*Comparison of Mean Bile Acid Ratios with those found in Sweden by Isaksson* (1954) *and Sjövall* (1960), *Using Normal Subjects*

| | Present results | | | |
|---|---|---|---|---|
| | Liver cancer | Control | Results of Isaksson | Results of Sjövall |
| G/T | 1·41 | 3·97 | .. | 3·20 |
| (GC + GD)/GCD | 1·07 | 0·93 | 1·38 | 1·70 |
| GD/GC | 0·47 | 0·46 | 0·36 | 0·55 |
| GD/GCD | 0·30 | 0·21 | 0·37 | 0·60 |

due to bacterial action as discussed above, or to effects of terminal malnutrition on liver function.

Table I shows that the liver cancer group comprised younger cases than the control group, and showed larger bile volumes, with smaller concentrations of total soluble matter, relatively larger amounts of insoluble matter, and rather smaller proportions of total measured components in the soluble matter (mean = 58·7%, compared with 66·4% for the controls). The cholesterol and phospholipid results were similar in liver cancer and control groups, and the mean cholesterol/phospholipid ratio of 0·14 resembled the figure of 0·11 found by Nakayama and Johnston (1962) for gall-bladder bile from normal Americans.

The mean ratio of total glyco- to total tauro-acids (G/T ratio) was 3·97 in the control group, therein resembling the normal ratio reported by Sjövall (Table II), but only 1·41 in the liver cancer group. This difference between the two African groups was highly significant, with $p < 0{\cdot}001$, and was the basis of the lower values for individual glyco-acids and higher values for tauro-acids in the liver cancer group. The G/T ratio was also below normal in the two cases with cancer of the urinary bladder (G/T = 0·48, 0·51), but not in the two cases with other types of cancer (G/T = 3·40, 6·00). Apart from the conditions discussed here, a low biliary G/T ratio also occurs in normal infants (Sjövall, 1959*b*) and in some cases of obstructive jaundice, though not portal cirrhosis (Ekdahl and Sjövall, 1957 ; Sjövall, 1960). For bile acids synthesized by homogenates of liver biopsies, the same ratio is low in hepatitis, obstructive jaundice and post-operative trauma (Ekdahl and Stenram, 1958).

The ratios between the bile acids themselves, apart from their conjugation, are best considered in terms of ratios between the corresponding glyco-acids, as taurochenodeoxycholic and taurodeoxycholic acids were not separated. The ratio of the synthesis of the 3$\alpha$,7$\alpha$,12$\alpha$-trihydroxyacid glycocholic acid (GC) to that of the 3$\alpha$,7$\alpha$-dihydroxyacid glycochenodeoxycholic acid (GCD) indicates the extent to which precursors of the latter acid are 12-hydroxylated in the liver to give precursors of the former acid (Bergström, 1959). Part of the glycocholic acid is further transformed into the 3$\alpha$,12$\alpha$-dihydroxyacid glycodeoxycholic acid (GD), which arises in the bile after 7-dehydroxylation of cholic acid by gut micro-organisms, absorption from the gut, reconjugation and re-excretion (Bergström, 1959). The relative extent of cholic acid synthesis is thus indicated most accurately by the ratio (GC + GD)/GCD.

This ratio was similar in the liver cancer and control groups (mean = 1·07 and 0·93 respectively—Table I), but the latter ratio was reduced to 0·71 ± 0·41 on omitting a single exceptionally high value of 2·29, and the ratio for the liver cancer group was then just not significantly above that of the controls ($0{\cdot}05 < p < 0{\cdot}10$). A comparable but more definite rise in the biliary ratio of tri- to di-hydroxy-acids was previously observed (Mirvish and Gillman, 1960) on feeding rats with dimethylaminoazobenzene (rat bile has very little deoxycholic acid).

The (GC + GD)/GCD ratios for both liver cancer and control groups were considerably less than those found in Sweden (Table II), and in only two cases did the ratio exceed the mean value of 1·70 reported by Sjövall. In portal cirrhosis Sjövall (1960) found low ratios of 0·4–1·1, and the ratio of trihydroxy- to dihydroxy-bile acids in serum is also depressed in this disease (Rudman and Kendall, 1957 ; Carey, 1958 ; Osborn, Wootton, Da Silva and Sherlock, 1959). The single case of liver cirrhosis without cancer in the present study showed a (GC + GD)/GCD ratio of 0·61. In the liver cancer group, this ratio could not be correlated with the degree of the liver cirrhosis present in all but one case. The low value of this ratio in the Africans thus suggests a prevalence of a liver state resembling that associated in Europeans with cirrhosis, in which 12-hydroxylation is apparently reduced. It may be relevant that Bersohn and Wayburne (1956) have demonstrated general liver dysfunction amongst Africans in South Africa, using standard liver function tests.

The similarity in the ratio of glycodeoxycholic to glycocholic acid (GD/GC) between the liver cancer, control and Swedish cases indicates that the Africans showed a normal conversion of the latter to the former acid by gut micro-organisms, and an active entero-hepatic cycle, even in the presence of liver cancer. The low ratio in the Africans of glycodeoxycholic to glycochenodeoxycholic acid (GD/GCD) presumably reflects the low GC/GCD ratio.

SUMMARY

Bile was collected post-mortem from the gall bladders of Africans in Mozambique. Bile extracts from 26 cases, including ten of primary liver cancer and two of urinary bladder cancer, were analysed for pH, cholesterol, phospholipid and the various conjugated bile acids. A new method is described for extracting cholesterol from bile extracts and bile. The ratio of glyco- to tauro-acids was lowered in the liver cancer and urinary bladder cancer cases to one third of the normal values, but this effect is not specific, as it has also been reported in several other conditions. The ratio (GC + GD)/GCD, which measures the extent of cholic acid synthesis relative to that of chenodeoxycholic acid, showed some tendency to be higher in the liver cancer than the control group, but was considerably less in both groups than in normal subjects examined in Sweden. The latter observation may indicate a general incidence amongst Africans of a liver state resembling that associated in Europeans with cirrhosis. In most other respects, the bile composition of the liver cancer and control groups were closely similar.

I wish to thank Professor J. Gillman of our Department and Dr. M. Prates of the Miguel Bombarda Hospital, Lourenco Marques, for carrying out the post-mortem examinations and bile collections, and for examining the liver sections ; Professor Gillman for his kind advice and encouragement ; Dr. E. Lieban of the Kaplan Hospital, Rehovoth, Israel, for supplying the biles used to check the extractions ; Dr. J. Sjövall for showing me his bile acid techniques ; and Messrs. N. G. N. Matthews and J. Makunga for excellent technical assistance. This work was supported by a grant from the National Cancer Association of South Africa, to whom thanks are expressed.

### REFERENCES

BERGSTRÖM, S.—(1959) ' The Biosynthesis of Terpenes and Sterols '. A Ciba Foundation Symposium. London (J. A. Churchill and Co.), p. 185.

BERSOHN, I. AND WAYBURNE, S.—(1956) *Amer. J. clin. Nutr.*, **4**, 117.

CAREY, J. B.—(1958) *J. clin. Invest.*, **37**, 1494.

EKDAHL, P. H. AND SJÖVALL, J.—(1957) *Acta chir. scand.*, **114**, 439.

*Idem* AND STENRAM, U.—(1958) *Ibid.*, **115**, 189.

FAILEY, R. B., BROWN, E. AND HODES, M. E.—(1960) *Arch. Path.*, **70**, 358.

FOLDES, F. F. AND WILSON, B. C.—(1950) *Analyt. Chem.*, **22**, 1210.

GLICK, D.—(1934) *J. Lab. clin. Med.*, **19**, 1012.

HAWK, P. B., OSER, B. L. AND SUMMERSON, W. H.—(1954) ' Practical Physiological Chemistry '. New York (McGraw-Hill) p. 590.

IRVIN, J. L., JOHNSTON, C. G. AND KOPALA, J.—(1944) *J. biol. Chem.*, **153**, 439.

ISAKSSON, B.—(1954) *Acta Soc. Med. Upsaliensis*, **59**, 307.

KUTTNER, T. AND LICHTENSTEIN, L.—(1932) *J. biol. Chem.*, **95**, 661.

MIRVISH, S. AND GILLMAN, J.—(1960) *Brit. J. Cancer*, **14**, 346.

NAKAYAMA, F. AND JOHNSTON, C. G.—(1962) *J. Lab. clin. Med.*, **59**, 365.

OSBORN, E. C., WOOTTON, I. D. P., DA SILVA, L. C. AND SHERLOCK, S.—(1959) *Lancet*, ii, 1049.

PRATES, M. D.—(1961) *Acta Un. int. Cancr.*, **17**, 718.—(1962) *Ibid.*, **18**, 643.

RUDMAN, D. AND KENDALL, F. E.—(1957) *J. clin. Invest.*, **36**, 530.

SCHOENHEIMER, R. AND SPERRY, W. M.—(1934) *J. biol. Chem.*, **106**, 745.

SJÖVALL, J.—(1955) *Ark. Kemi*, **8**, 317.—(1959*a*) *Clin. Chim. Acta*, **4**, 652.—(1959*b*) *Ibid.*, **4**, 793.—(1960) *Ibid.*, **5**, 33.